# Supplementary material for: The MODY-associated TALK-1 L114P mutation causes islet α-cell overactivity and β-cell inactivity resulting in transient neonatal diabetes and glucose dyshomeostasis in adults
Source: bioRxiv. 2023 Jul 26:2023.06.20.545631. Preprint. [Version 2] doi: 10.1101/2023.06.20.545631 (PMC10401960; doi:10.1101/2023.06.20.545631)
Supplement: Supplement 1 [file media-1.pdf]

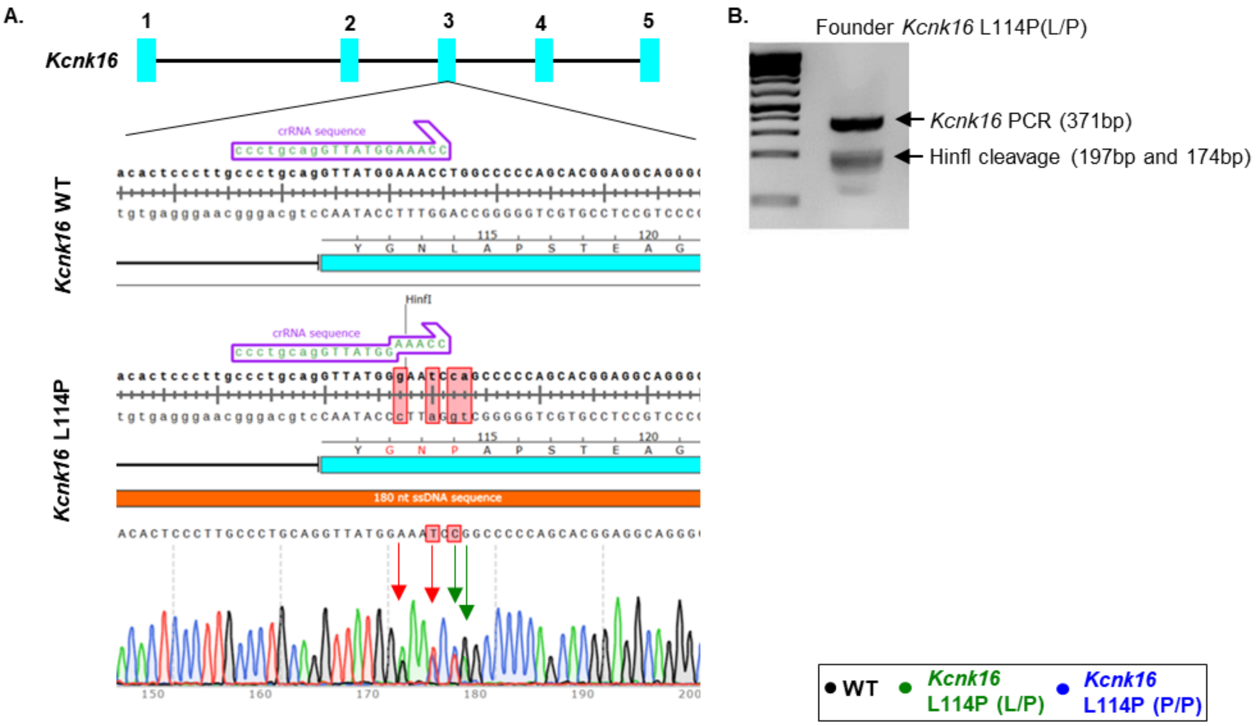

C.

Genotype	Expected	Observed
Kcnk16 WT	25%	27%
Kcnk16 L114P(L/P)	50%	53%
Kcnk16 L114P(P/P)	25%	6.3%

$\chi^2 = 6.648$, $P = 0.036$

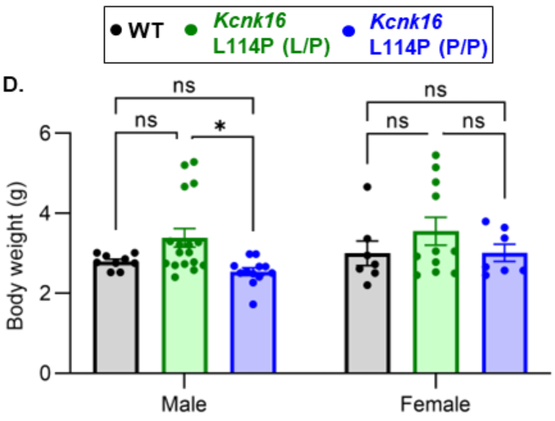

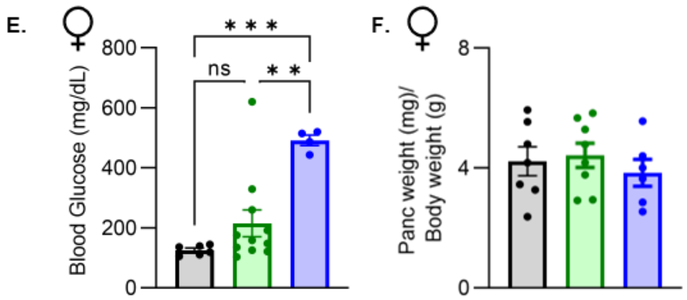

Supplemental figure 1. Generation of *Kcnk16* L114P model and assessment of neonatal glucose homeostasis and lethality. A. Targeted region of *Kcnk16* exon 3 using CRISPR/Cas9 leading to the introduction of HinfI restriction enzyme site (red arrows) and CTG to CCA mutation in codon 337 corresponding to p.TALK-1 L114P (green arrows). B. PCR confirmation of the male founder *Kcnk16* L114P (L/P) mouse using HinfI

restriction digestion. C. χ^2 analysis of the F1 progeny from B6; CD-1 *Kcnk16* L114P (L/P) crosses. D. Body weight measurements of male (left) and female (right) wildtype (WT; black), heterozygous *Kcnk16* L114P (L/P; green), and homozygous *Kcnk16* L114P (P/P; blue) mice on P4. E. Blood glucose measurements of female mice on P4. F. Pancreas weight/ body weight measurements of P4 female mice. Data are presented as mean±SEM. Data were analyzed using student's t-test or one-way ANOVA.

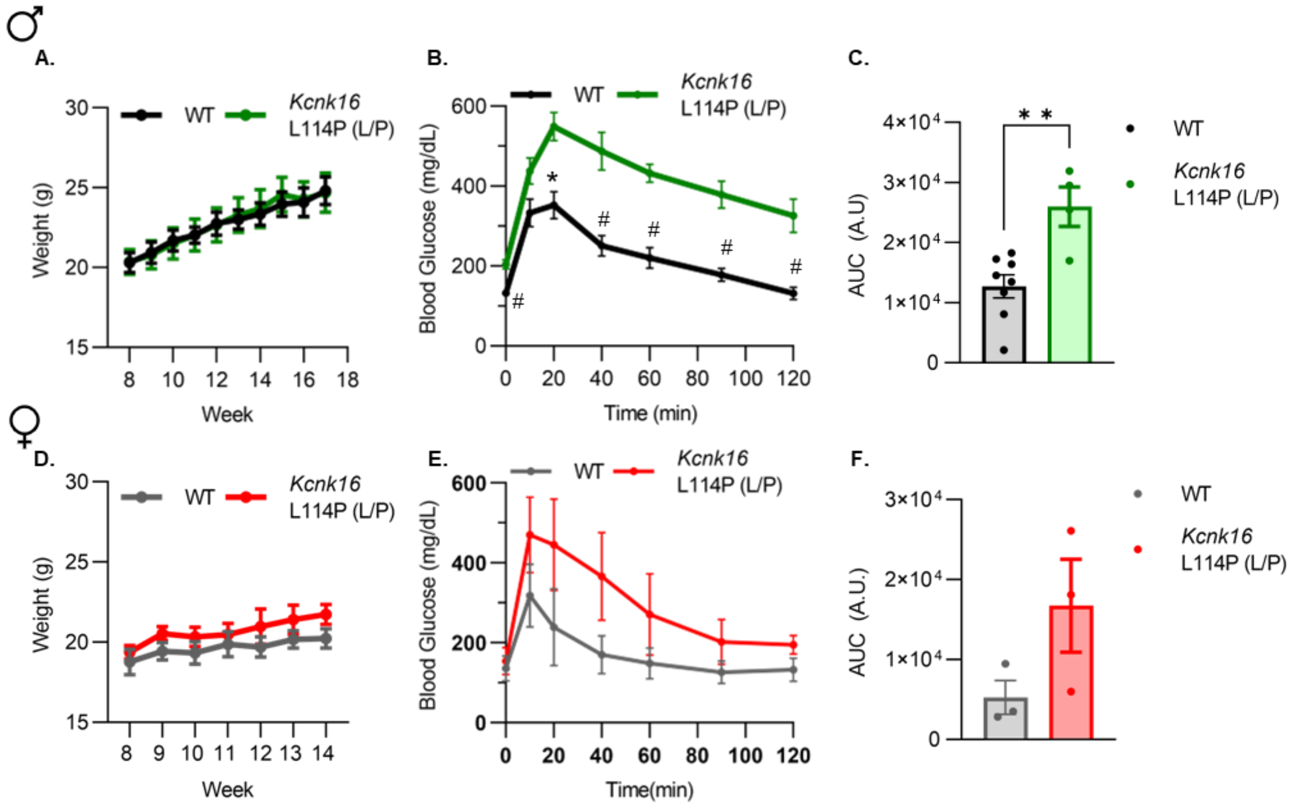

Supplemental figure 2. Glucose homeostasis is also impaired in the *Kcnk16* L114P (L/P) mice in the B6 background. A. Body weight measurements of male WT (black; N=8) and *Kcnk16* L114P (L/P; green; N=4) mice in the C57Bl/6J background. B. Intraperitoneal glucose tolerance test (i.p. GTT) performed in 10-week-old male mice following a 4-hour fast in response to 2mg/g glucose injection. C. Average AUC of the 2-hr GTT excursion profiles in (c). D. Body weight measurements of female WT (gray; N=3) and *Kcnk16* L114P (L/P; red; N=3) mice. E. I.P. GTT performed in 11-week-old female mice following a 4-hour fast in response to 2mg/g glucose injection. F. Average AUC of the 2-hr GTT excursion profiles in (E). Data are presented as mean±SEM. Data were analyzed using student's t-test; **P<0.01, #P<0.001.

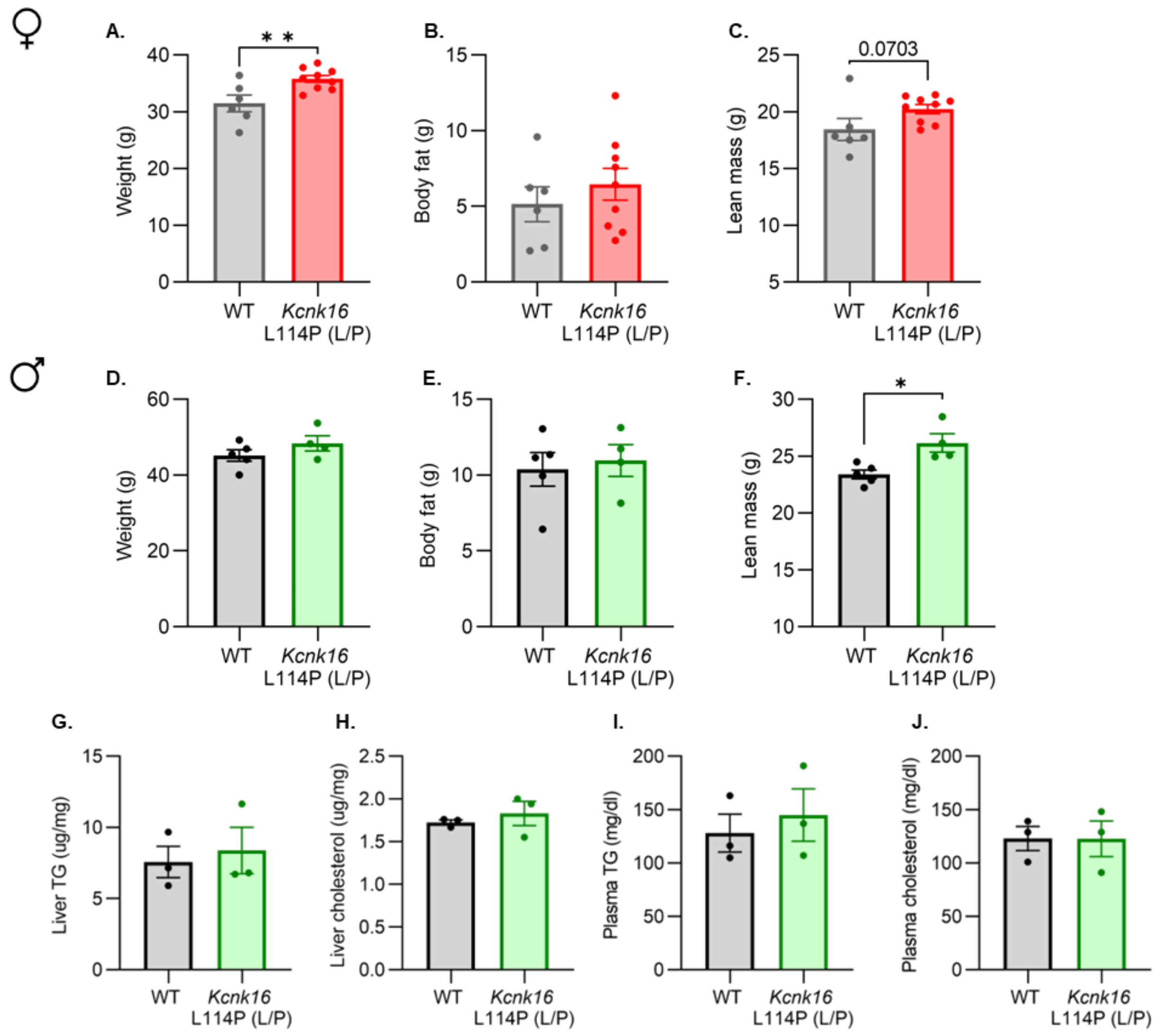

Supplemental figure 3. Body composition measurements and assessment of plasma and liver triglycerides and total cholesterol. A.-F. Body composition analysis of male and female B6; CD-1 WT and *Kcnk16* L114P (L/P) mice assessing weight (g), body fat (g), and lean mass (g) (N=5-9 mice/genotype). G.-J. Average liver and plasma cholesterol and triglyceride levels in male B6; CD-1 WT and *Kcnk16* L114P (L/P) mice (N=3/genotype). Data are presented as mean±SEM. Data were analyzed using student's t-test.

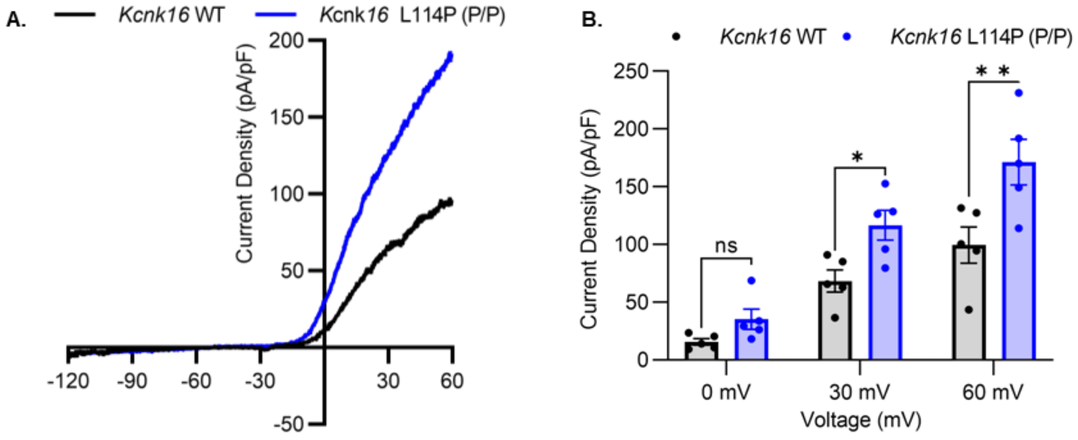

Supplemental figure 4. K2P currents in β-cells from homozygous *Kcnk16* L114P (P/P) mice also exhibit a modest increase. A. Representative whole-cell K2P current density (pA/pF) recorded using a voltage ramp (-120 mV to +60 mV) in 11 mM G in islets from B6; CD-1 WT and *Kcnk16* L114P (P/P) P4 neonates. B. Average current density (pA/pF) measured at the specified membrane potentials (N=5 cells/genotype). Data are presented as mean±SEM. Data were analyzed using two-way ANOVA.

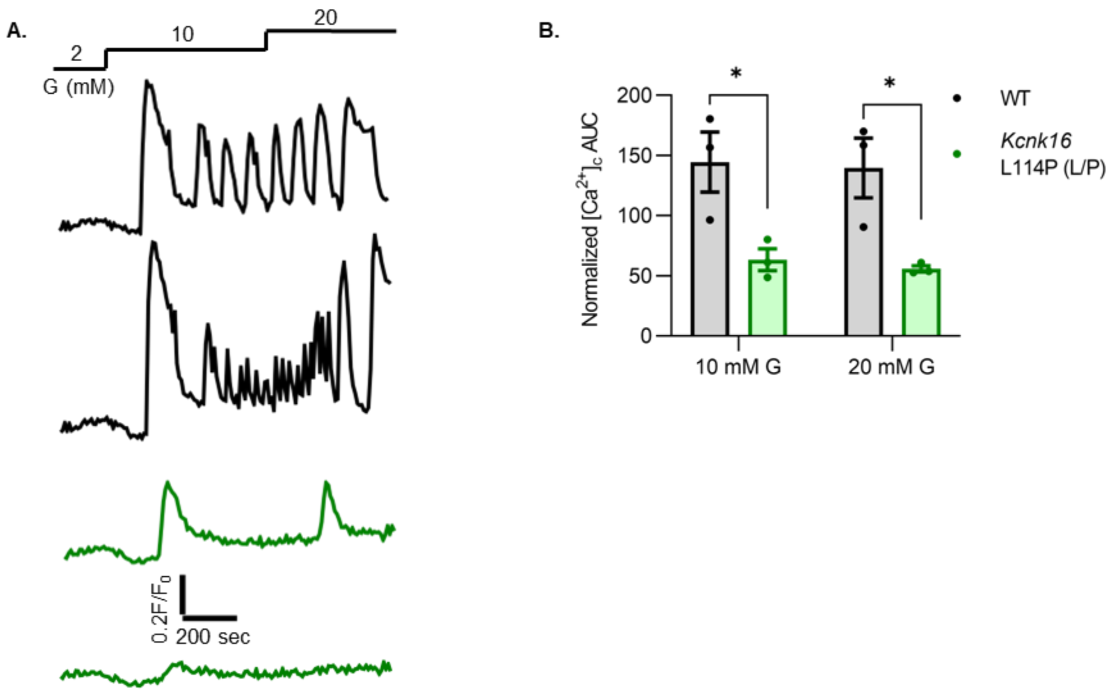

Supplemental figure 5. Islets from *Kcnk16* L114P (L/P) mice on the B6 background also exhibit blunted glucose-stimulated Ca^{2+} entry. A. Representative GSCI traces in islets from male WT and *Kcnk16* L114P(L/P) mice in the C57BL/6J background in response to 2 mM G, 10 mM G, and 20 mM G (N=3 mice/genotype). B. Average total AUC in response to the indicated glucose concentrations in islets from male WT and *Kcnk16* L114P(L/P) mice. Data are presented as mean±SEM. Data are analyzed using student's t-test.

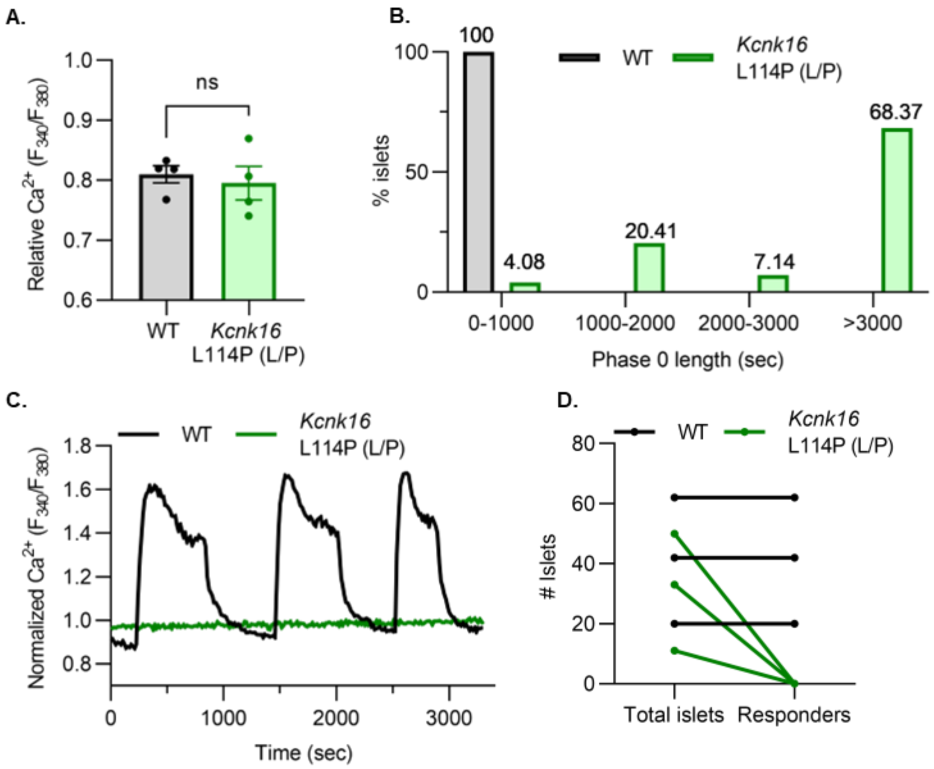

Supplemental figure 6. *Kcnk16* L114P (L/P) islets exhibit prolonged glucose-stimulated phase 0 $[Ca^{2+}]_{ER}$ uptake and show a complete absence of Ca^{2+} oscillations. A. Average relative $[Ca^{2+}]_c$ at 2 mM G in islets from B6; CD-1 WT and *Kcnk16* L114P (L/P) mice (N=4 mice/genotype). B. Percent islets that exhibit the corresponding phase 0 response length (sec) in WT and *Kcnk16* L114P (L/P) mice. C. Representative glucose-stimulated $[Ca^{2+}]_c$ oscillations recorded at 9 mM G in islets from WT and *Kcnk16* L114P (L/P) mice. D. Total number of islets analyzed for $[Ca^{2+}]_c$ oscillations vs. the number of islets that exhibited $[Ca^{2+}]_c$ oscillations from WT and *Kcnk16* L114P (L/P) mice (N=3 mice/genotype). Data are presented as mean±SEM. Data are analyzed using student's t-test.

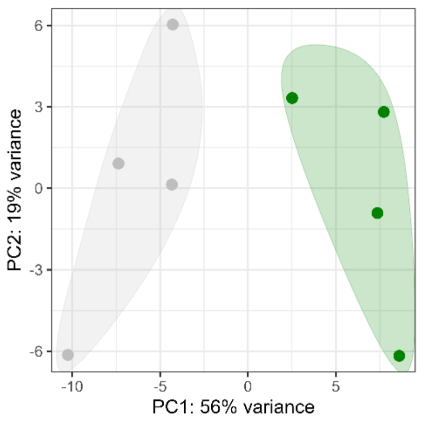

Supplemental figure 7. Principal component analysis (PCA) showing clustering of WT (gray) and *Kcnk16* L114P (L/P; green) islet RNA samples.

Gene	Forward primer	Reverse primer
18sRNA	GTAACCCGTTGAACCCCATT	CCATCCAATCGGTAGTAGCG
Cacna1g	GAGACACAGAGTACGGGAGC	CAGGCATTTCATGGTCAGCG
Sst	CCACCGGGAAACAGGAACTG	TTGCTGGGTTCGAGTTGGC
Asb11	TGGTGGACTGTCAGACTGCT	ATTGACGTTGATGCCTTGCG
Fxyd3	ACTCTGCTTTCTCCCGGAAC	CTCGGAGGCTGTACCAATCATA
Aldh1a3	GGGTCACACTGGAGCTAGGA	CTGGCCTCTTCTTGGCGAA
Camk1d	CCGCCCTACAGCATTAGTCT	GAAAAGGCCCCAGTTCCGA
Cxcl1	ACCCAAACCGAAGTCATAGCC	TTGTCAGAAGCCAGCGTTCA
Adcyap1r1	CTGCGTGCAGAAATGCTACTG	AGCCGTAGAGTAATGGTGGATAG
Aldob	AGAAGGACAGCCAGGGAAAT	GTTCAGAGAGGCCATCAAGC
Pdk4	TGGTAGCAGTAGTCCAAGATGC	GTGGATTGGTTGGCCTGGAA
Tgfb2	TCGACATGGATCAGTTTATGCG	CCCTGGTACTGTTGTAGATGGA

Supplemental table 1. Mouse primer sequences used for qRT-PCR.
